# Multi-level advances in databases related to systems pharmacology in traditional Chinese medicine: a 60-year review

**DOI:** 10.3389/fphar.2023.1289901

**Published:** 2023-11-14

**Authors:** Mengyue Fan, Ching Jin, Daping Li, Yingshan Deng, Lin Yao, Yongjun Chen, Yu-Ling Ma, Taiyi Wang

**Affiliations:** ^1^ Innovation Research Institute of Chinese Medicine, Shandong University of Traditional Chinese Medicine, Jinan, China; ^2^ Northwestern Institute on Complex Systems, Northwestern University, Evanston, IL, United States; ^3^ College of Acupuncture and Massage, Shandong University of Traditional Chinese Medicine, Jinan, China; ^4^ Oxford Chinese Medicine Research Centre, University of Oxford, Oxford, United Kingdom

**Keywords:** TCM databases, systems pharmacology, formula-ZHENG relationship, complex biological system, network analysis

## Abstract

The therapeutic effects of traditional Chinese medicine (TCM) involve intricate interactions among multiple components and targets. Currently, computational approaches play a pivotal role in simulating various pharmacological processes of TCM. The application of network analysis in TCM research has provided an effective means to explain the pharmacological mechanisms underlying the actions of herbs or formulas through the lens of biological network analysis. Along with the advances of network analysis, computational science has coalesced around the core chain of TCM research: formula-herb-component-target-phenotype-*ZHENG*, facilitating the accumulation and organization of the extensive TCM-related data and the establishment of relevant databases. Nonetheless, recent years have witnessed a tendency toward homogeneity in the development and application of these databases. Advancements in computational technologies, including deep learning and foundation model, have propelled the exploration and modeling of intricate systems into a new phase, potentially heralding a new era. This review aims to delves into the progress made in databases related to six key entities: formula, herb, component, target, phenotype, and *ZHENG*. Systematically discussions on the commonalities and disparities among various database types were presented. In addition, the review raised the issue of research bottleneck in TCM computational pharmacology and envisions the forthcoming directions of computational research within the realm of TCM.

## 1 Introduction

Chinese herbal medicines have primarily originated from foods. Over long periods of practical living experience, the medicinal properties of many herbs were gradually established (Hou and Jiang, 2013; Gu and Pei, 2017). Subsequently, foods with therapeutic properties were progressively separated and designated for specialized use as medicines (Hou and Jiang, 2013; He et al., 2018; Long et al., 2022). Human foraging practices frequently entail the amalgamation of various food sources, a tendency that has played a significant role in the creation of Traditional Chinese Medicine (TCM) formulas (Hou and Jiang, 2013). From its inception, TCM may have involved the application of herbal combinations. Some of these herbal combinations were stable and clearly effective and were therefore documented and passed down as formulas through generations. This resulted in the creation of over 300,000 known formulas (Li et al., 2008), laying the foundation for clinical TCM treatments. However, pharmacological research of TCM formulas faces the significant challenge of analyzing combinations of 100 or more chemical compounds (which are also named components) per formula (Zhao et al., 2010). Statistics on the total amount of targets corresponding to each compounds in PubChem Bioassays database is 3.7 in average (Jalencas and Mestres, 2013; Hu et al., 2014). According to the number mentioned above, a given TCM formula could potentially regulate over 370 targets. Thus, the “one drug-one target” pharmacological research methodology is insufficient to explain the therapeutic effects and mechanisms of action associated with TCM formulas (Ding et al., 2020).

Deciphering the intricate pharmacological mechanisms associated with herbs and formulas is a monumental task for researchers in the field of TCM (Wang et al., 2021b; Li et al., 2022b). Due to the “black box” nature of complex biological systems, studies of formula efficacy would do well to take a more macroscopic approach (Yao et al., 2013; Huang et al., 2023), i.e., research needs be designed using a “system-to-system” framework for clinical and pharmacological investigations of entire formula instead of disassembling formulas and studying the components (Liang et al., 2012). This approach involves observing the relationships between formulas (input) and effects in biological systems (output). The research philosophy behind chemical drug development is fundamentally guided by reductionism, with antagonism serving as a primary principle (Saks et al., 2009). The key paradigm of drug discovery revolves around the creation of inhibitors or activators that specifically target particular molecular entities (Jendza et al., 2019; Gong et al., 2023). Over time, this approach has proven imperfect due to the discovery of off-target responses, which may have toxicological impacts or cause other side effects. Given the extensive range of enzymatic systems, classes, and isoforms that have been identified in biological systems, the development of many target-specific agents has relied on trial-and-error methodologies (Méndez-Lucio et al., 2016; Paydas, 2019). However, regulation of targets by formulas does not always require an extremely high level of specificity, and exceptionally high activity levels may not be necessary (Méndez-Lucio et al., 2016). Formulas themselves constitute complex systems, wherein synergistic interactions between components can lead to optimal effects to maximize impacts on the human biological systems (Chen et al., 2018). Research of the pharmacology associated with specific formulas therefore necessitates unveiling (or partially) of the “black box” that is synergistic interactions between components and their interactions with the human biological system. This requires accurate simulation of the alterations that occur in various nodes within the biological system due to regulation by a specific formula (Tan et al., 2019). The goal is to establish correlations (and ideally quantitative relationships) between changes in a formula and changes in clinical phenotypes.

Advances in computational biochemical analyses have ushered in a new age of TCM research (Barabási et al., 2011; Wang et al., 2022a). Cooperative regulation of multiple targets by multi-component medicine is an effective strategy for altering the output of complex systems (Csermely et al., 2005; Zhang et al., 2014; Ramsay et al., 2018). Mathematical models that reflect complex systems are exceptionally potent tools in systems biology research (Kitano, 2002; Liu and Barabási, 2016; Zhao et al., 2019). The advent of artificial intelligence (AI), particularly deep learning, has allowed the accumulation of TCM data with unprecedented depth and complexity (Chen et al., 2019). Studying a substantial number of effective formulas (rather than individual formulas) using phenotypes or clinical manifestations as outputs can allow elucidation of the intricate relationships among formulas, herbs, components, targets, phenotypes, and *ZHENG*. Computer science is a powerful tool that facilitates TCM research by allowing both establishment of relationships and large-scale collection of relevant data (Zhang et al., 2019a). However, it is crucial to exercise caution in utilizing such tools to ensure that results are grounded in reality. The establishment of trustworthy, accurate TCM databases will thus be a pivotal step in unraveling the complexities of herbs or formulas (Saks et al., 2009).

**FIGURE 1:**
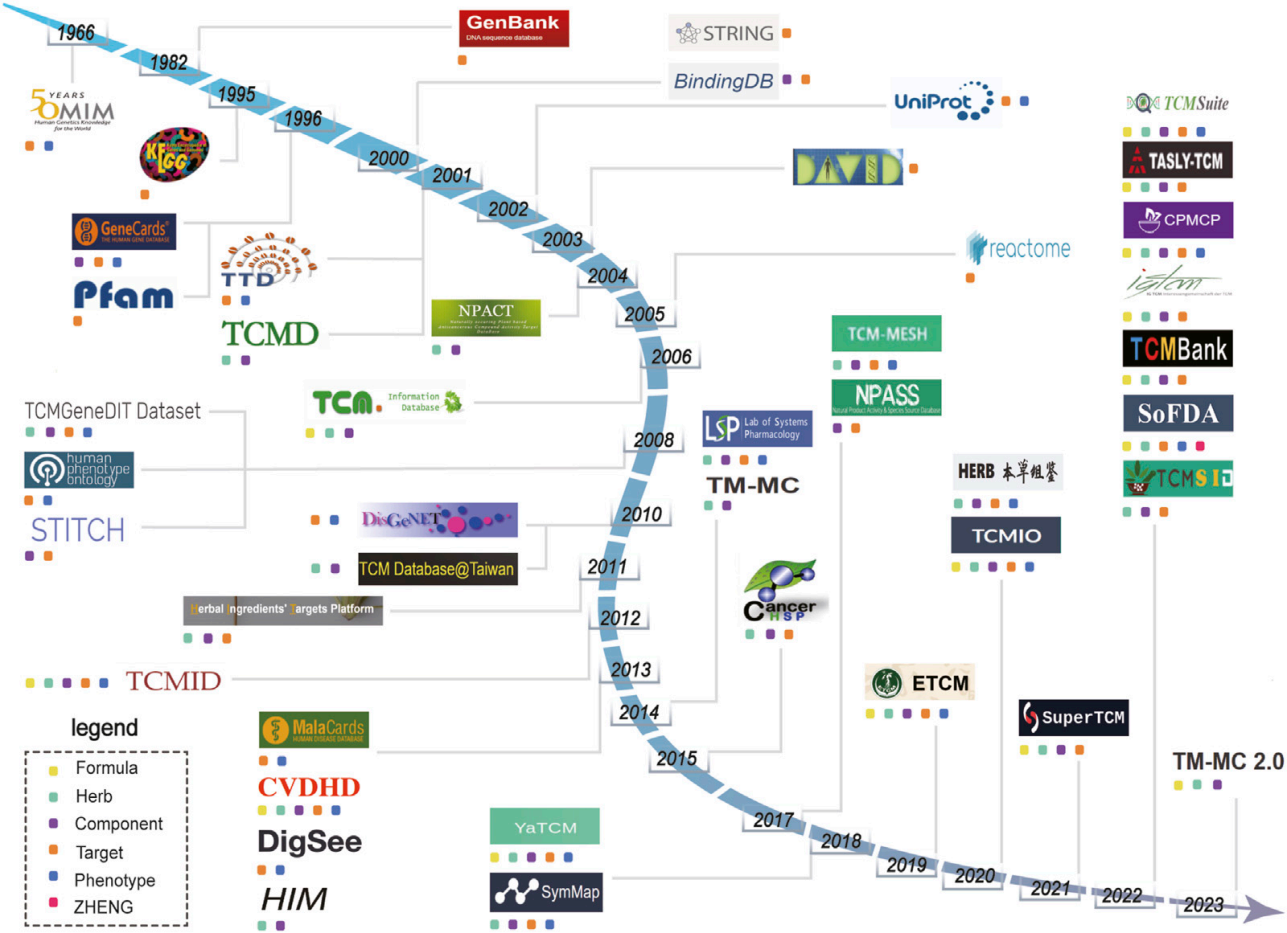
Timeline showing the establishment of databases related to traditional Chinese medicine (TCM) from 1966 to 2023. Different colored squares represent the main entity data contained in the database. The yellow squares represent “Formula”. The green squares represent “Herbs”. The purple squares represent “Components”. The orange squares represent “Target”. The blue squares represent “Phenotype”. The red squares represent “*ZHENG*”.

Research into the pharmacology underlying TCM necessitates the accumulation of extensive data for multiple parts of the TCM system: formulas, herbs, components, targets, phenotypes, and *ZHENG* (Han et al., 2017). Clear delineations of various relationships (e.g., formula-component, component-target, and target-phenotype relationships) are vital (Xu et al., 2021; Zhao et al., 2023b; Gan et al., 2023). Since 1960, databases have been developed and are now available for use in computational TCM research (Figure 1). In this review, we conduct a retrospective examination of the establishment of these databases, with a particular emphasis on comparison based on the inclusion of formula, herb, component, target, biological function, phenotype, and *ZHENG* data. We aim to consolidate and analyze the relationships between various entities within these databases, including formula-component, component-target, target-phenotype, and phenotype-*ZHENG* relationships. This review summarizes the trends, identifies gaps in the existing research, and suggests directions for future development of the databases related to systems pharmacology in TCM.

## 2 TCM formula databases

Initially, TCM formulas included only a small number of herbs. The herbs were consistently combined and administered in fixed proportions, which were documented and transmitted over time (Zha et al., 2015). With the evolution of medical practices, current formulas encompass not only ancient formulas but also modern empirical formulas and commercially prepared Chinese patent medicines. These formulas comprise the fundamental data within TCM formula databases, and there is a total of 21 such databases. These databases primarily contain fundamental information such as formula compositions, therapeutic functions, indications, and methods of use. Of the 21 databases, 18 are academic and 3 are commercial (Table 1). TCM-ID was one of the earliest TCM formula databases which brings the concept of formula-herb-component-target relationships in the form of databases (Chen et al., 2006). Chinese Medicine Think Tank (a big data analysis platform for TCM) houses the largest collection of TCM formula resources, including ∼300,000 formulas (Chinese Medicine Think Tank-a big data analysis platform for TCM, 2017). Over the Counter TCM Database and the Database of Standardized TCM Chinese Patent Drugs focus on marketed TCM formulas (Database of Standardized for Chinese patent drugs, 2017; OTC Chinese Herbal Medicine Database, 2017). These databases provide more comprehensive records of Chinese patent Drugs. DRUGDATAEXPY and Pharnexcloud are the major databases used in the pharmaceutical industry (DRUGDATAEXPY, 2009; Pharnexcloud, 2021). In addition to providing extensive formula resources, they also enable queries related to research, clinical trials, marketing, production inspections, and sales of specific formulas.

**TABLE 1:** List of TCM formula databases.

Database name	Type	Number of formulas	Data source	Presentation modules directly related to TCM formula	Analysis modules directly related to TCM formula
TCM-ID Chen et al. (2006) (http://tcm.cz3.nus.edu.sg/group/tcm-id/tcmid.asp)	Academic database	7443 formulas	ChP-TCM, TCM formulas approved by China food and drug administration	Related herb, indication, reference	Targeted human proteins by the components of formula, targeted pathogenic microbes by the components of formula, GO associated to targeted human proteins, KEGG pathways associated to targeted human proteins, target expression in healthy human tissues
YaTCM Li et al. (2018a) (http://cadd.pharmacy.nankai.edu.cn/yatcm/home)	Academic database	1813 formulas	Literatures, ancient books, ChP-TCM (2015), TCMID	Related herb, reference, traditional explanation, traditional usage, traditional/modern application	Network and pathways analysis
TCMID 2.0 Huang et al. (2018) (http://www.megabionet.org/tcmid/)	Academic database	99582 formulas	Encyclopedia of TCM, literatures	Related herb, related formula component, use method, indication, reference	
TCMIO Liu et al. (2020) (http://tcmio.xielab.net)	Academic database	1493 formulas	ChP-TCM (2015)	Reference, efficacy, indication	Pathway enrichment analysis, network visualization
TCMIP v2.0 Wang et al. (2021a) (http://www.tcmip.cn/)	Academic database	3959 formulas	ETCM	Related herb, dosage form, administration, formula type, related *ZHENG*, indication, related target, related disease	GO analysis, reactome pathway, target prediction and functional analysis of formulas, analysis of medication rules in formulas, reverse searching for formulas based on enrichment analysis
LTM-TCM Li et al. (2022a) (http://cloud.tasly.com/#/tcm/home)	Academic database	48126 formulas	TCMID, TCM-ID, ChP-TCM, TCM typical formula database, NMPA protected traditional medicines, NMPA OTC database (TCM), NMPA national basic drug database (TCM), national service platform for academic experience of famous TCM doctor	Related herb, related symptom, reference, related component	The knowledge graph of prescript with related herbs and symptoms, statistics of components related to the formula, formula similarity analysis, formula gene target search and prediction, enriched pathways, formula target genes enrichment analysis
CPMCP Sun et al. (2022) (http://cpmcp.top)	Academic database	1469 Chinese patent drugs, 656 formulas	Compendium of national standards for Chinese patent medicines, Chinese medicine works	Related herb, related TCM symptom, related MM symptom, related component, related target, related disease, reference	Visualization of the following entities/associations: formula-TCM symptom-MM symptom-disease, formula-herb-component-target-disease
TCM-Suite Yang et al. (2022) (http://TCM-Suite.AImicrobiome.cn)	Academic database	6692 formulas	Database	Related herb, related component, related protein, related disease	Visualization of the following entities/associations: formula-herb-component
ETCM v2.0 Zhang et al. (2023) (http://www.tcmip.cn/ETCM2/front/#/)	Academic database	48442 formulas, 9872 Chinese patent drugs	Ancient books, ChP-TCM (2020)	Related herb, formula type, related symptom/sign, dosage form, related disease, efficacy, source, related component, related target, related enriched disease, related ZHENG, indication, quantitative information of marker component	Number distribution of components hitting the candidate targets, functional enrichment analysis of targets, feature distribution of components, expression heatmap of targets, similar formulas, similar Chinese patent drugs, network visualization
TM-MC 2.0 TM-MC 2.0, 2023 (https://tm-mc.kr/index.jsp)	Academic database	5075 formulas	Ancient books	Related herb, source, indication	
CNKI-TCM formula knowledge base CNKI-TCM formula knowledge base, (2023) (https://kb.tcm.cnki.net/TCM/TCM/Index?dbcode=ZYFF)	Academic database	Over 20000 formulas	Ancient books	Source, efficacy, use method, related herb, TCM song tip, indication, medical record	
Modern Application Database of Herbal Formulations Modern Application Database of Herbal Formulations, (2017) (http://cintmed.cintcm.com/cintmed/searchIndex/basic.html?dbtype=xdfj&pdh=15)	Academic database	9651 formulas	ChP-TCM, drug standards issued by the ministry of health-Chinese patent drugs	Source, efficacy, use method, related herb, dosage form, use method, related component, contraindication, adverse reaction, pharmacological action, clinical application, toxicological experiment, physicochemical property, manufacturer	
Database of Standardized for Chinese patent drugs Database of Standardized for Chinese patent drugs, (2017) (http://cintmed.cintcm.com/cintmed/searchIndex/basic.html?dbtype=zyfj&pdh=20)	Academic database	4052 Chinese patent drugs	Drug standards issued by the ministry of health - Chinese patent drugs (1989-1998)	Source, efficacy, use method, alias, related herb, dosage form, use method, contraindication, quality requirement, specification, storage, standard number	
Chinese Herbal Formulas Database Chinese Herbal Formulas Database, (2017) (http://cintmed.cintcm.com/cintmed/searchIndex/basic.html?dbtype=zyfj&pdh=14)	Academic database	85174 formulas	Literatures, Ancient books	Contraindication, pharmacological action, source, efficacy, use method, alias, related herb	
TCM Ancient Formulas Database TCM Ancient Formulas Database, (2023) (http://zyygx.cintcm.ac.cn:9698/zyygx/)	Academic database	Over 160000 formulas	Ancient books	Related syndrome, efficacy, use method	
OTC Chinese Herbal Medicine Database OTC Chinese Herbal Medicine Database, (2017) (https://cintmed.cintcm.com/cintmed/searchIndex/basic.html?dbtype=xdfj&pdh=21)	Academic database	2852 formulas	OTC Chinese herbal medicine released by the Chinese government (1999)	Source, efficacy, use method, alias, related herb, dosage form, type, contraindication, specification, adverse reactions, standard number, storage, quality requirement, pharmacological action	
Chinese Medicine Formulae Image Database Chinese Medicine Formulae Image Database, (2023) (https://library.hkbu.edu.hk/electronic/libdbs/cmfid/index.html)	Academic database	182 formulas	Commonly used Chinese formulas	Type, application, source, efficacy, related herb, use method	Introduction to formulas with illustrations
Chinese Medicine Think Tank- a big data analysis platform for TCM Chinese Medicine Think Tank- a big data analysis platform for TCM, (2017) (https://www.duguji.cn/login)	Commercial database	300000 formulas	Ancient books, the empirical formula of famous veteran teran doctors of TCM	Source, efficacy, related herb, use method	
DRUGDATAEXPY DRUGDATAEXPY, (2009) (https://db.yaozh.com/fangji)	Commercial database	34214 formulas	Drug standards issued by the ministry of health - Chinese patent drugs, ancient books	Dosage form, contraindication, indication, application, efficacy, related herb	
Pharnexcloud Pharnexcloud, (2021) (https://www.pharnexcloud.com/)	Commercial database	35000 formulas	ChP-TCM, ancient books, the empirical formula of famous veteran teran doctors of TCM	Indication, contraindication, source, efficacy, related herb, use method	
Huabing data platform Huabing data platform, (2023) (http://www.huabeing.com/pres/index)	Commercial database	200000 formulas		Source, efficacy, related herb, related disease, related symptom	Medication rules analysis in formulas compositions, the core function of auxiliary discovery in formulas, the auxiliary confirmation of the core components of formula

TCM, traditional Chinese medicine; GO, Gene ontology; OTC, over the counter; ChP-TCM, pharmacopoeia of the people’s Republic of China; MM, modern medicine.

In 2016, with the emergence of databases like BATMAN-TCM that allow for customized predictive analysis of the pharmacological mechanisms of TCM formulas, databases for TCM were no longer limited to the functions of browsing and searching (Liu et al., 2016). The development of database functions became more geared towards analysis and prediction, and it was only then that TCM formula databases began to be utilized in a truly meaningful way. The principles of herbal compatibility are crucial for the effectiveness of TCM formulas. Formula databases not only allow for direct retrieval of herbal composition of formulas but also enable algorithmic-based analysis of the patterns governing herbal combinations. Through analysis of existing TCM formulas, CPMCP has summarized frequently used herbal combinations in TCM clinical practice. This functionality has helped to uncover the habitual pairings and contraindications between various herbs, shedding light on principles of herbal compatibility (Sun et al., 2022). Huabing data, an intelligent TCM big data platform, enables screening of disease-related formulas based on input herbal combinations. It dynamically calculates and analyzes the top 20 herbs, symptoms, diseases, and functions related to the treatment of certain diseases using formulas present in the database (Huabing data platform, 2023). TCMIP allows the selection of target TCM formula groups based on criteria such as formula composition and primary diseases treated. It calculates the frequency of herb usage, herb properties, inter-herb associations, and the frequency of formula targets within a selected formula group (Wang et al., 2021a). This approach facilitates innovative research of TCM formulas. For example, researchers have constructed a scoring system for the post-effects of drug combinations based on formula-herb relationships. The scoring system is utilized to recommend the most effective herbal combinations for certain diseases (Niu et al., 2023). The use of analytical algorithms on data from these databases can accelerate explorations of the intricate networks underlying TCM formula efficacy (Wang et al., 2021b).

One effective approach to simplifying the study of the pharmacological mechanisms of formulas is to make use of databases such as, ETCM, TCMIP, LTM-TCM, TCM-ID, which enable direct prediction of the pharmacological mechanisms of formulas (Chen et al., 2006; Xu et al., 2019; Wang et al., 2021a; Li et al., 2022a; Zhang et al., 2023). However, it's important to note that the quality of data and the dimensions covered vary among these databases, which can impact the accuracy of predictive results. Further evaluation is necessary for the data in these databases. Besides of that dosage and usage have varied significantly between regions and dynasties (Zha et al., 2015). Therefore, the results of the verification of the usage and dosage of individual herbs within formulas, as well as the sources of this information, should also be indispensable data within the database. The dosage of an herb significantly determines the concentrations of its components within the human body fluid and then may impacts the activity of component’s targets, which would be the key logic of both wet and dry experiments. However, most databases have not effectively cleaned dosage-related data during the inclusion of herbal formulas and data cleaning processes. Systematic research of ancient texts is essential to methodically organize and standardize ancient formulas. This highlights the needs of establishing uniform, rigorous standards, and quantitative dosage information for TCM data (Wang et al., 2021b).

## 3 Herb databases

Ancient Chinese people, through extensive medical practices over a long period of time, experimented with many medicinal substances derived from animals, plants, minerals, microorganisms, and other sources (Wang et al., 2017). These substances were documented, and their functions continuously verified over time. At present, TCM herbs encompass plant-based medicines, animal-derived medicines, and mineral-based medicines, with plant-based medicines being the predominant category. Herb databases are commonly used to compile fundamental information about herbs, such as properties, meridians, regions of usage, flavors, effects, and indications. This information came from various sources, including the Pharmacopoeia of the People’s Republic of China (ChP-TCM), the fourth national survey on Chinese Materia Medica Resources, books, literatures, and dictionaries. A total of 24 databases related to herbs have been identified, 21 of which are academic and 3 of which are commercial (Table 2). The Pharmacloud database contains the largest number of herb resources at −18,000 (Pharnexcloud, 2021). TCMID holds the distinction of being the academic database with the most extensive collection of herb resources, encompassing a total of 10,846 (Xue et al., 2012). China’s multi-ethnic composition means that various ethnic groups have discovered numerous herbs rooted in their own cultural practices. To enhance drug development, databases related to herbs from different ethnic groups have also been established, including databases for Tibetan, Mongolian, Uyghur, and Yao medicine.

**TABLE 2:** List of herb databases.

Database name	Type	Number of herbs	Data source	Presentation modules directly related to herb	Analysis modules directly related to herb
TCM Database@Taiwan Chen, (2011) (http://tcm.cmu.edu.tw/)	Academic database	453 herbs	Chinese medical texts and dictionaries	Type, related component	
TCMSP Ru et al. (2014) (http://sm.nwsuaf.edu.cn/lsp/tcmsp.php)	Academic database	499 herbs	ChP-TCM	Related component, related target, related disease	Network representation of component-target interactions, network representation of target-disease interactions
TM-MC 2.0 TM-MC 2.0, 2023 (https://tm-mc.kr/index.jsp)	Academic database	635 herbs	Literatures	Related component	
CancerHSP Tao et al. (2015) (http://lsp.nwsuaf.edu)	Academic database	2439 anticancer herbs	Literatures	Related component	
TCM-Mesh Zhang et al. (2017b) (http://mesh.tcm.microbioinformatics.org/)	Academic database	6235 herbs		Property, meridian, use part, efficacy, indication, related component, related disease, related research, related target, toxicity and side effect	
TCMID 2.0 Huang et al. (2018) (http://www.megabionet.org/tcmid/)	Academic database	10846 herbs	TCM-ID, books, literatures	Property, meridian, use part, efficacy, indication, related component	Herb-target-disease network
YaTCM Li et al. (2018a) (http://cadd.pharmacy.nankai.edu.cn/yatcm/home)	Academic database	6220 herbs	TCMID, TCMSP, TCM Database@Taiwan, books, literatures	Picture, efficacy, indication	Herb-herb network
TCMIO Liu et al. (2020) (http://tcmio.xielab.net)	Academic database	618 herbs	ChP-TCM (2015)	Species, family, use part, property, flavor, meridian, efficacy, indication	Formula-herb-component-target network, pathway enrichment analysis
TCMIP v2.0 Wang et al. (2021a) (http://www.tcmip.cn/)	Academic database	402 herbs	ETCM	Picture, type, description in Chinese/English, habitat, collection time, appearance, specification, property, flavor, meridian, indication, related disease, related formulas, chemical profiling and the corresponding candidate target gene list, quality control	GO analysis, reactome pathway, target prediction and functional analysis of herb, exploration and functional analysis of molecular mechanisms related to TCM medicinal properties, network mining in TCM
SuperTCM Chen et al. (2021) (http://tcm.charite.de/supertcm)	Academic database	6516 herbs	Books, databases	Use part, picture, related component, related target, related disease, related recipe	KEGG metabolic pathways
HERB Fang et al. (2021) (http://herb.ac.cn/)	Academic database	7263 herbs	SymMap, TCMID, TCMSP, TCM-ID	Use part, efficacy, related component, related target, related disease, related high-throughput experiment, ID mapping of TCMID database	
LTM-TCM Li et al. (2022a) (http://cloud.tasly.com/#/tcm/home)	Academic database	9122 herbs	TCMID, CancerHSP, TCMIP, ChP-TCM (2020)	property, herb toxicity, use part, herb smell, related formula	The knowledge graph of herb with related prescripts and components, summary of herb query results (number of TCM formulas containing this herb, number of components, number of herb’s target, number of components supported by literature evidence, count of literature evidence), ADME of the herb component, herb-component target prediction, target gene set enrichment
HIT 2.0 Yan et al. (2022) (http://hit2.badd-cao.net)	Academic database	1250 herbs	Literatures	Efficacy, related target, related component, ID mapping of TCM-ID database	
CPMCP Sun et al. (2022) (http://cpmcp.top)	Academic database	1560 herbs	ChP-TCM	Property, meridian, type, use part, related Chinese patent drug, related formula, related TCM symptom, related MM symptom, related component, related target, related disease, ID mapping of HERB, SymMap databases	Relationship network (herbs-Chinese patent drugs-formulas-TCM symptom-MM symptom-component-target-disease), the common compatibility of medicinal materials
TCMSID Zhang et al. (2022a) (https://tcm.scbdd.com)	Academic database	499 herbs	TCMSP, SymMap	Category, picture, related component	Automatically calculate and generate the network analysis diagram of herb-component-target-drug relationship
IGTCM Ye et al. (2023) (http://yeyn.group:96/)	Academic database	83 herbs		Related component, efficacy, indication	
SymMap v2 Wu et al. (2019) (http://www.symmap.org/)	Academic database	698 herbs	ChP-TCM (2020)	Property, meridian, class, use part, efficacy, ID mapping of TCMID, TCM-ID, TCMSP databases	Overview of the herb-component network, overview of the herb-*ZHENG*-TCM symptom network, functional enrichments of genes
TCM-Suite Yang et al. (2022) (http://tcm-suite.aimicrobiome.cn/)	Academic database	7322 herbs	Database	Property, meridian, efficacy, indication, use part, related formula, related component, related protein, related disease	Visualization of the following entities/associations: formula-herb-component-protein
ETCM v2.0 Zhang et al. (2023) (http://www.tcmip.cn/ETCM2/front/#/)	Academic database	2079 herbs	The fourth national survey on Chinese materia medica resources, ChP-TCM (2020), authoritative Chinese medical books and dictionaries	Family, habitat, use part, herb classification based on efficacy, property, flavor, meridian, indication, related Chinese patent drug, related formula, related target, related enriched disease, related component	Number distribution of components hitting the candidate targets, functional enrichment analysis of targets, feature distribution of components, expression heatmap of targets, similar herbs (based on components, targets), herb-component network
TCMBank Lv et al. (2023) (https://TCMBank.cn/)	Academic database	9192 herbs	TCM Database@Taiwan	Related components, property, efficacy, related disease, related gene, ID mapping of HERB, Timebank, TCM-ID databases	Relationship map (herb-components-targets-diseases), AI-assisted drug-drug interactions prediction model to produce the prediction results of the mutual exclusion of Chinese-Western medicine
The Chinese traditional medicine database The Chinese traditional medicine database, (2017) (https://cintmed.cintcm.com/cintmed/searchIndex/basic.html?dbtype=xdfj&pdh=5)	Academic database	8173 herbs	ChP-TCM, authoritative Chinese medical books and dictionaries	Property, toxicity and side effect, quality control, efficacy, processing method, use part, indication, family, habitat	
Huabing data platform Huabing data platform, (2023) (http://www.huabeing.com/pres/index)	Commercial database		Ancient books	Related component, related symptom, related disease, related herb, efficacy	
Pharnexcloud Pharnexcloud, (2021) (https://www.pharnexcloud.com/)	Commercial database	18000 herbs		Type, related formula, related component, efficacy, habitat	
DRUGDATAEXPY DRUGDATAEXPY, (2009) (https://db.yaozh.com/fangji)	Commercial database	2000 herbs		Habitat, efficacy	

TCM, traditional Chinese medicine; AI, artificial intelligence; ChP-TCM, pharmacopoeia of the people’s Republic of China, GO, gene ontology; MM, modern medicine.

The relationships between herbs and active components are currently key areas of focus in herb research (Fu et al., 2014; Zeng et al., 2022b). These linkages are included in herb databases. Active components are not only the primary materials that compose herbs but are also crucial for their therapeutic effects (Liu C. et al., 2018a). In 2018, following an update to the TCMID, there was a significant improvement in the coverage of herbs (Huang et al., 2018). Additionally, it introduced mass spectrometry (MS) data for these herbs, which served the purpose of distinguishing differences in the quality of herbs. Quantitative data for the characteristic components in each herb, as specified in the ChP-TCM, are available in the, ETCM and TCMIP databases (Wang et al., 2021a; Zhang et al., 2023). SymMap annotates components in four categories based on experimental MS data from ChP-TCM and from the literatures: quality control components, blood components, metabolite components, and other components (Wu et al., 2019). Utilizing herb-component relationship information from such a database, it is possible to construct more intricate features for herbs. This can be achieved, for example, by building heterogeneous herb-component-target networks. Such efforts enhance the accuracy of intelligent formula recommendation systems based on deep learning, such as FordNet (Zhou et al., 2021). Herb-component-target relationships in these databases also enable researchers to measure the effectiveness of specific herbs in treating diseases. This approach can then be used to identify herbs that are highly associated with specific diseases based on the importance of a particular target within a disease network (Wang et al., 2021d; Niu et al., 2023). For the identification of biological components in TCM, TCM-Suite gathered sequences and associated information for six marker genes: ITS2, matK, trnH-psbA, trnL, rpoC1, and ycf1 (Yang et al., 2022). Therapeutic efficacy of herbs is associated with the components and its content in the herb. A counterpart example in compound chemical drug is that there are fixed usage ratios for the synergistic effects of components (Ferrannini et al., 2022). For example, a fixed-ratio combination of insulin glargine and lixisenatide can better control the blood sugar levels in patients with diabetes (Aroda et al., 2016). Inappropriate ratios can lead to opposite effects (Létinier et al., 2023). In the context of components in herbs, the same principle holds true. Therefore, establishment of the herb-component relationships also requires the critical quantitative information-the content of components in herbs (Heinrich et al., 2022). Currently, there is a substantial accumulation of research on the identification and content measurements of components in herbs, including high-performance liquid chromatography, high-performance liquid chromatography-MS, and gas chromatography-MS, etc. (Arrizabalaga-Larrañaga et al., 2021; Papatheocharidou and Samanidou, 2023), but there is still a lack of databases for comprehensive aggregation and compilation of quantitative research data on components in herbs.

## 4 Component databases

Regardless of whether a so-called “herb” of interest is a plant, animal product, mineral-based medicine, the active components of which are chemical substances. Herbal component databases include information about the chemical components that have been extracted or isolated from single herbs or formulas. Such databases source their data from the literature, experimental data, and/or preexisting databases, encompassing essential details such as chemical structure, and CAS registry number of component. A total of 28 databases related to chemical components in TCM herbs have been identified (Table 3).TCM-Suite has the largest number of TCM chemical components at 704321 components, but it only 54,868 herb-component relationships (Yang et al., 2022). Some of these databases are more specialized: TCMIO, CancerHSP, and NPACT primarily focus on collecting information about active components related to tumors, whereas CVDHD contains data about active components associated with cardiovascular diseases (Mangal et al., 2013; Tao et al., 2015; 2015; Liu et al., 2020). Databases for herb components specifically offer a wealth of resources for modern drug development (Fu et al., 2016; Zhang et al., 2023).

**TABLE 3:** List of component databases.

Database name	Number of components	Data source	Presentation modules directly related to component	Analysis modules directly related to component
TCMD He et al. (2001) (http://repharma.pku.edu.cn/tcmd.html)	23033 components	Book: TCM: molecular structures, natural sources, and applications	Chemical structure, physicochemical property	
TCM-ID Chen et al. (2006) (http://tcm.cz3.nus.edu.sg/group/tcm-id/tcmid.asp)	5669 components	Books, literatures	Chemical structure, CAS	
TCM Database@Taiwan Chen, (2011) (http://tcm.cmu.edu.tw/)	20000 components	Chinese medical texts and dictionaries, Medline, ISI Web of knowledge	Chemical structure, molecular volume, molecular properties (ALogP, molecular polar surface area, number of hydrogen bond acceptors, number of hydrogen bond donors, number of rotatable bonds)	
CVDHD Gu et al. (2013) (http://pkuxxj.pku.edu.cn/CVDHD)	35230 compounds	CHDD, UNPD	Chemical structure, CAS, molecular properties (AlogP, number of hydrogen bond acceptors, number of hydrogen bond donors, number of rotatable bonds), docking results, cardiovascular-related disease, pathway and clinical biomarker	
NPACT Mangal et al. (2013) (http://crdd.osdd.net/raghava/npact/)	1574 bio- compounds	PubMed, literatures	Chemical structure, properties (physical, elemental, and topological), cancer type, cell lines, inhibitory values (IC50, ED50, EC50, GI50), related target, commercial supplier, *in-vitro* anticancer activity, *in-vivo* anticancer activity, CAS, ID mapping of PubChem database	Drug-likeliness filters, similarity search
TCMID 2.0 Huang et al. (2018) (http://www.megabionet.org/tcmid/)	43413 total components, 1045 formula components	TCM-ID, HIT, TCM Database@Taiwan, books, literatures	Chemical structure, related target, related herb, ID mapping of PubChem database	Component-targets network, component-targets-drug-disease network
HIM Kang et al. (2013) (http://www.bioinformatics.org.cn/)	361 components		*In-vivo* metabolism information, bioactivity, organ/tissue distribution, toxicity, ADME, clinical research profile	
TCMSP Ru et al. (2014) (http://sm.nwsuaf.edu.cn/lsp/tcmsp.php)	29384 components	PubChem, ChEMBL, ChemSpider	Related target, ADME, Chemical structure, CAS, related disease, related target, related herb, ID mapping of PubChem database	
CancerHSP Tao et al. (2015) (http://lsp.nwsuaf.edu)	3575 anticancer compounds	Books, literatures, PubMed, Google scholar, PubChem, ChemSpider	Pharmacological property, molecular property, anticancer activity, related target, related herb, reference, ADME	
BindingDB Gilson et al. (2016) (https://www.bindingdb.org/rwd/bind/index.jsp)	490000 compounds	ChEMBL, PubChem, UniChem, ZINC	Chemical structure, ligand, substrate, IC50, citation, inhibitor, related target	Protein-small molecule affinity
STITCH Szklarczyk et al. (2016) (http://stitch.embl.de)	430000 compounds		Chemical structure, related target	Protein-chemical interaction networks
TCMAnalyzer Liu et al. (2018b) (http://www.rcdd.org.cn/tcmanalyzer)	16437 components	TCMSP, TCMID	Chemical structure	Substructure search, scaffold search, 2D similarity search, 3D similarity search
ETCM v2.0 Zhang et al. (2023) (http://www.tcmip.cn/ETCM2/front/#/)	38298 components	ChP-TCM (2020), literatures	Chemical structure, molecular weight, physicochemical property, pharmacokinetic property, toxicology, related formula, related herb, related target	
TCM-Suite Yang et al. (2022) (http://TCM-Suite.AImicrobiome.cn)	704321 components	Database	Chemical structure, related herb, related formula, related protein, related disease	Visualization of the following entities/associations: formula-herb-component-protein-disease
LTM-TCM Li et al. (2022a) (http://cloud.tasly.com/#/tcm/home)	34967 components	TCM-ID, TCMID, TCM-Mesh, TCMIP, TM-MC	Basic physical and chemical information, ADME, chemical structure, related herb, related formula, related target	Enriched signal pathways, component target genes enrichment analysis
TCMIO Liu et al. (2020) (http://tcmio.xielab.net)	16437 components	TCMAnalyzer, TCMSP, TCMID	Chemical structure, synonyms, related target	Herb-component-target network
TM-MC 2.0 TM-MC 2.0, 2023 (https://tm-mc.kr/index.jsp)	34081 components	Literatures	Chemical structure, related herb, related target, number of hydrogen bond acceptors, number of hydrogen bond donors, number of rotatable bonds	
HIT 2.0 Yan et al. (2022) (http://hit2.badd-cao.net)	1237 components	TCM-ID, PubChem, ChEMBL	Chemical structure, CAS, related target, related herb, ID mapping of ChEMBL, PubChem, TCM-ID databases	Automatic target-mining and my-target curation from daily released PubMed literatures
SymMap Wu et al. (2019) (http://www.symmap.org/)	19595 components	TCMID, TCMSP, TCM-ID	OB score, type, CAS, related herb, related *ZHENG*, related TCM symptom, related MM symptom, related target, related disease, ID mapping of PubChem, TCMID, TCM-ID, TCMSP databases	
SuperTCM Chen et al. (2021) (http://tcm.charite.de/supertcm)	55772 components	Databases, books	Chemical structure, related target, related KEGG pathway, related disease, related herb, related recipe, ID mapping of PubChem, ChEMBL databases	Herb-component relationships, component-target relationships, component-target-pathway relationships, component-target-disease relationships
HERB Fang et al. (2021) (http://herb.ac.cn/)	49258 components	SymMap, TCMID, TCMSP, TCM-ID	Chemical structure, related herb, ID mapping of TCMID database	Paper mined target genes, paper mined diseases, differentially expressed genes were calculated from high-throughput experiments deposited in the GEO database, GO enrichment analysis were performed based on the differentially expressed genes, KEGG enrichment analysis were performed based on the differentially expressed genes, Connectivity analysis was performed by mapping the differentially expressed genes of herb/component to CMap touchstone datasets, using the CMap website
TCMBank Lv et al. (2023) (https://TCMBank.cn/)	61966 components	TCM Database@Taiwan	Related herb, related target, related disease, OB score, molecular polar surface Area, number of hydrogen bond acceptors, number of hydrogen bond donors, number of rotatable bonds, C count, O count, CAS, ID mapping of PubChem, TCMID, TCM-ID, TCMSP, SymMap databases, chemical structure	Relationship map (herb-component-target-disease)
CPMCP Sun et al. (2022) (http://cpmcp.top)	26341 components	TCMID, TCMSP, TCM-ID	OB score, CAS, related formula, related herb, related TCM symptom, related MM symptom, related target, related disease, ID mapping of PubChem, SymMap databases	Component-herb-TCM symptom-MM symptom-target-disease relationship network
TCMSID Zhang et al. (2022a) (https://tcm.scbdd.com)	20015 components	Literatures, TCMSP, SymMap	CAS, chemical structure, synonyms, ADME, related target, ID mapping of PubChem database	Herb-component-target-drug multilevel interaction network, component structural reliability evaluation, component structural classification
NPASS Zhao et al. (2023a) (http://bidd.group/NPASS)	43285 compounds	PubMed, PubChem, ZINC, ChEMBL, BindingDB, TCM-ID, TCM Database@Taiwan, TCMID, TCMSP, TM-MC, StreptomeDB, HerDing, TTD, ChEMBL, DrugBank, IUPHAR/BPS	Chemical classification, related herb, chemical structure, natural product quantity composition/concentration, ADMET, chemically structural similarity, biological similarity, related target, synonym, synthetic gene cluster, physical and chemical property, ID mapping of PubChem, ChEMBL database	
TCMIP v2.0 Wang et al. (2021a) (http://www.tcmip.cn/)	7284 components		Chemical structure, ALogP, LogD, molecular solubility, molecular volume, molecular surface area, molecular polar surface area, number of hydrogen bond acceptors, number of hydrogen bond donors, number of rotatable bonds, ADMET, drug likeness weight, drug likeness grading, related target, related disease, CAS, reference, related herb, related formula, ID mapping of PubChem, ChEMBL databases	Herb-component-formula network
IGTCM Ye et al. (2023) (http://yeyn.group:96/)	1033 components		Chemical structure, OB score, external link (KEGG), reference	
Chinese Traditional Medicine Chemical Components Database Chinese Traditional Medicine Chemical Ingredients Database, (2017) (https://cintmed.cintcm.com/cintmed/searchIndex/basic.html?dbtype=xdfj&pdh=7)	27593 components	Books, literatures	Chemical structure, physicochemical property, pharmacokinetic property, clinical application	

TCM, traditional Chinese medicine; ChP-TCM, pharmacopoeia of the people’s republic of China; GEO, gene expression omnibus; GO, gene ontology; MM, modern medicine; OB, oral bioavailability.

The relationships between components and targets represent a key link connecting two intricate systems: herbs and human biological systems (Stitziel and Kathiresan, 2017). Data mining and computational chemistry approaches are currently being used to collect and organize known component-target relationships and to predict and validate previously unknown component-target relationships (Chen et al., 2016). Several component databases TCM provide both information about known component-target relationships and functionalities for predicting such relationships. The HIT and HERB databases contain information about component-target relationships obtained through text mining of the literature (Fang et al., 2021; Yan et al., 2022). HIT categorizes component-target relationships component into three types: “Directly inhibit/activate,” “Indirectly inhibit/activate,” and “Enzyme substrate”. Users can refer to the associated literature to learn more about specific component-target relationships. More importantly, HIT facilitates automatic target mining and curation of “My-target” information from newly released PubMed literature (Yan et al., 2022).

For components lacking reported relationships with a target, several computational chemistry approaches have been significantly developed. These approaches include ligand-based methods, target-based methods, and target-ligand methods, all of which aim to predict relationships between components and proteins (Sadybekov and Katritch, 2023). The SwissTargetPrediction is a widely used web tool, available online since 2014, designed to predict the most probable protein targets of small molecules. Predictions are made using the similarity principle through reverse screening. In the latest updated version, the models have been recalculated, achieving a success rate of at least one correct human target in the top 15 predictions for more than 70% of external compounds (Daina et al., 2019). BindingDB is a database that focuses on relationships between small molecules and their corresponding targets. The BindingDB website provides specialized tools that leverage its extensive data collection, allowing researchers to generate hypotheses for protein targets of a given bioactive component or to predict components that are bound by a particular protein. Additionally, the website offers virtual component screening using methods like maximal chemical similarity, binary kernel discrimination, and support vector machines(Gilson et al., 2016). To meet the demand for predicting targets of components, component databases have also started incorporating target prediction functionality. SysDT is a model that was designed to predict potential targets of components within the TCMSP database (Ru et al., 2014). SysDT has demonstrated remarkable predictive performance for drug-target relationships (Yu et al., 2012). ETCM v2.0 uses a target identification method that is based on a two-dimensional ligand similarity search module within the D3CARP platform and utilizes data from Binding DB (Zhang et al., 2023). To enhance the accuracy of target prediction, TCMSID employs multiple target prediction methods, including similarity ensemble approach, SwissTargetPrediction, HitpickV2, PPB, PPB2, and CHEMBL (Zhang al., 2022a). LTM-TCM integrates component-target information from various sources, including the BATMAN-TCM, ChEMBL, and STITCH databases. LTM-TCM retains target scores from different sources to enable personalized target screening based on user-defined thresholds (Li et al., 2022a).

The systematic collection and organization of herb components in databases forms the foundation of target prediction to decipher the multiple pharmacological actions of a given compound. Target prediction methods have the potential to significantly shorten drug development timelines, but the accuracy of computational studies remains relatively low. In practice, even the most successful virtual screening campaigns typically result in only 10%–40% of candidate hits being confirmed through experimental validation (Sadybekov and Katritch, 2023). A multitude of virtual screening efforts produced predominantly discouraging outcomes. For instance, the antimalarial drug ebselen, which had been identified through an early virtual screening process, ultimately proved unsuccessful in clinical trials (Sadybekov and Katritch, 2023). Therefore, it is essential to conduct more comprehensive *in vitro* and *in vivo* studies and develop improved methods for evaluating the above results. These results recorded in online databases should also have clear indications of their sources, to aid researchers in assessing the reliability of the data.

## 5 Target and target-related biofunction databases

Targets are the smallest functional units within an organism, serving as the internal nodes of complex systems (Turkarslan et al., 2014). They carry out various functions in numerous pathways and phenotypic responses, acting as bridges between medicines and the human biological system (Pfister and Ashworth, 2017; Santos et al., 2017). Drug mechanisms of action involve interactions between components and their targets. The initial paradigm in this area posited that a single component would act on a single target (Koeberle and Werz, 2014). However, further research revealed that nearly all natural and human-synthesized components interact with multiple targets (Plazas et al., 2022). Target databases primarily encompass genetic and protein-related information. Existing types of target databases include drug target databases, disease target databases, and specific target databases. These databases typically include basic information such as the target type, function, and origin, which are often sourced from the literature. UniProt, NCBI, and GeneCards are examples of target databases that provide comprehensive genetic and protein sequence information along with functional details (Table 4) (Safran et al., 2010; Brown et al., 2015; The UniProt Consortium, 2023). ETCM, TCMID, YaTCM, HIT, HERB, DisGeNET, and other databases also include information about targets, but these primarily focus on the relationships between targets and components or diseases (Piñero et al., 2017; Li et al., 2018a; Huang et al., 2018; Huang et al., 2018; Xu et al., 2019; Fang et al., 2021; Yan et al., 2022; Zhang et al., 2023). They often therefore have a decreased emphasis on the functional details of targets. Researchers have used target databases for purposes such as analysis of target-phenotype relationships (e.g., SymMap) (Wu et al., 2019; Lv et al., 2023). Target relationships in the TCMSP, TCMID, and TCM-ID databases have been used to map symptom-related genes and herb-related targets to human protein interaction networks (Chen et al., 2006; Xue et al., 2012; Ru et al., 2014; Huang et al., 2018). Through analysis of their topological relationships within a network, the distances between gene nodes can be calculated to infer distances between symptom modules, providing information about symptom co-occurrence and similarity. This approach has been employed to evaluate herb effectiveness for specific symptoms. It is a robust method for deciphering the mechanisms of herb and for predicting early-stage drug efficacy for diseases of interest (Gan et al., 2023).

**TABLE 4:** List of target databases.

Database name	Number of targets	Data source	Presentation modules directly related to target	Analysis modules directly related to target
TTD Li et al. (2018b); Zhou et al. (2022) (https://db.idrblab.net/ttd/)	3578 targets	Literatures	Target type, related disease, biochemical class, related drug, related regulator, related target profile in patient, related target affiliated biological pathway, related model, related study, ID mapping uniport database	All known drugs of a target are clustered based on multiple or single drug-like properties, which is displayed using the hierarchical clustering map, heatmap and bar plot, sequence similarity searching
NPASS Zhao et al. (2023a) (http://xin.cz3.nus.edu.sg/group/ttd/ttd.asp)	7753 targets		Target type, organism of target, biological activities of natural product against the target, related component, related specie	
Uniprot The UniProt Consortium, (2023) (https://www.uniprot.org/)	Over 227 million protein sequences, 451000 proteomes	Completely sequenced viral, bacterial, archaeal, and eukaryotic genomes	Function, subcellular location, related disease/variant, showing features for signal, chain, disulfide bond, glycosylation, expression (tissue specificity, gene expression database, organism-specific database), interaction (binary interaction, protein-protein interaction database, chemistry) structure, family/domain, sequence/isoform, similar protein	Find a protein sequence to run BLAST sequence similarity search by uniport ID, the align tool enables users to make multiple sequence alignments which can now be viewed in two ways-the wrapped view allows for a quick scan of the alignment, and the overview allows researchers to zoom in/out and move through the sequences in a user-defined manner, find uniport entries through parts of their peptide sequences, retrieve/ID mapping
Genecards Rebhan, (1997); Safran et al. (2010) (https://www.genecards.org/)	418808 entries, 43626 HGNC approved, 21702 protein coding, 292000 RNA genes	Databases	Genomic, protein, family/domain, function, localization, pathway/interaction, related drug, related component, transcript, expression, ortholog, paralog, variant, related disease, related product, reference	
Genbank Benson et al. (2018) (https://www.ncbi.nlm.nih.gov/genbank/)			Sources, feature, origin, reference	
Pfam Mistry et al. (2021) (http://pfam.xfam.org/)	19632 entries, 655 clans		Description, reference, protein, taxonomy, proteome, alphafold, pathway, genome 3D	

Biological pathways can be considered as subsystems within complex systems. They serve as a framework for conducting pharmacological TCM research. These pathways can provide explanations for the complex mechanisms that link herbs to physiological changes. They often play significant roles in elucidation of interactions between drugs and biological functions (Wang et al., 2022b). Many target function databases integrate information about genes and genomes with higher-level functional annotations (Zeeshan et al., 2020). These data can then be used to systematically analyze gene functions based on known biological processes in an organism. Such databases are thus commonly utilized in conducting gene functional enrichment analyses, pathway-related analyses, and protein-protein interaction analyses. KEGG is a reference database for biological interpretation of genome sequences and other high-throughput data. The primary functionalities for biological process analyses are biochemical pathway mapping, metabolic network construction, genome comparison and merging, and enzyme database construction for target molecules (Kanehisa et al., 2017). BioCyc compiles and references genomes and metabolic pathways from thousands of sequenced organisms (Karp, 2005). Reactome systematically generates ordered molecular transformation networks, resulting in formation of classical metabolic maps. This database also associates human proteins with their molecular functions, offering a resource that serves as both a record of biological processes and a tool for discovering new functional relationships from data such as gene expression levels or mutations in tumor cells. Additionally, it can predict target biological processes of ion channels (Jassal et al., 2019). DAVID database consists of six tools: the functional annotation clustering, the functional annotation chart, the functional annotation table, gene functional classification, gene ID conversion, and gene name batch viewer (Sherman et al., 2022). The STRING database is used for analysis of protein-protein interactions. Individual protein queries generate a network composed of all proteins that interact with the queried protein (von Mering et al., 2003). This is particularly valuable for exploring interactions among input proteins; for example, it can be used to analyze the connections among differentially expressed proteins identified from proteomic data (Szklarczyk et al., 2021).

## 6 Phenotype databases

From the perspective of a complex system, the state of an organism corresponding to any abnormal phenotype is an abnormal steady state (Tyler et al., 2016). Such an abnormal steady state entails multiple nodes balance within the system. Likewise, interventions should target several nodes simultaneously to effectively restore the system to its normal steady state. Phenotype databases primarily focus on collecting data related to diseases, symptoms, and other phenotype-related entities. These databases provide robust datasets for those researching the mechanisms underlying TCM efficacy, primarily sourced from the literature and from other databases. Currently, a total of 13 databases have been compiled that provide detailed descriptions of diseases and symptoms (Table 5). TCMBank is the most comprehensive repository of disease-related resources, encompassing 32,529 data points (Lv et al., 2023).

**TABLE 5:** List of phenotype databases.

Database name	Number of phenotypes	Data source	Presentation modules directly related to phenotype	Analysis modules directly related to phenotype
OMIM Amberger et al. (2015) (http://omim.org)	7894 phenotypes	Biomedical literatures	Gene-phenotype relationship, clinical synopsis, phenotypic series, description, clinical feature, cytogenetics, molecular genetics, reference; external links: protein, animal models, clinical resources	Graphical representation of phenotype/gene relationship
LTM-TCM Li et al. (2022a) (http://cloud.tasly.com/#/tcm/home)	1928 TCM symptoms		Related formula	Intelligent TCM formula based on existing formulas for the symptom, frequency of components in TCM formulas for the symptom, frequency of symptoms co-occurred with the given, the combination frequency of herb in formulas
HPO Köhler et al. (2021) (https://hpo.jax.org)	over 156000 annotations to hereditary diseases		Hierarchy, synonyms, reference, related gene, related disease	Profile search (allow you compare your profile across species with a specific set of genes or diseases)
DisGeNET Piñero et al. (2017) (http://www.disgenet.org)	30170 diseases	MeSH, OMIM, DO, ICD-9, ClinVar, GWAS Catalog, UniProt, GAD, BeFree, Wikipathways, ChEMBL, Gene Expression Atlas	Semantic type, phenotypic abnormality, disease ontology, summary of gene-disease association, evidence for gene-disease association, summary of variant-disease association, evidence for gene-disease association, summary of disease-disease association, disease mapping, ID mapping of UMLS, MeSH, OMIM databases	
MalaCards Rappaport et al. (2017) (http://www.malacards.org/)	20000 diseases	OMIM, GT, GR, GHR, Orphanet, NIH RD, Wiki, DISEASES, DO, NiNDS	Aliases/classification, related disease, related symptoms/phenotype, related drugs/therapeutics, genetic test, anatomical context, reference, related gene, variation, disease gene expression data, pathways, GO term	
SymMap Wu et al. (2019) (http://www.symmap.org/)	2518 ZHENGs/symptoms, 1148 MM symptoms, 14086 diseases	OMIM, MeSH, Orphanet, UMLS, MalaCards	*ZHENG* type, symptom locus, symptom property, symptom type, related MM symptom, related disease	
ETCM v2.0 Zhang et al. (2023) (http://www.tcmip.cn/ETCM2/front/#/)	8045 diseases	HPO, OMIM, DisGeNET, ORPHANET	Global category, anatomical category, related target	
TCMID 2.0 Huang et al. (2018) (http://www.megabionet.org/tcmid/)	4633 diseases, 2679 TCM diseases	OMIM	Description, clinical feature, related target	
TCMGeneDIT Fang et al. (2008) (http://tcm.lifescience.ntu.edu.tw/)	3360 diseases	PharmGKB	Related target	
SuperTCM Chen et al. (2021) (http://tcm.charite.de/supertcm)	8634 diseases	ICD-10-CM	ICD-10-CM detail, related component, related target, related herbs, related recipe	
HERB Fang et al. (2021) (http://herb.ac.cn/)	28212 diseases	Literatures, DisGeNET	Disease type, related component, related target, related herb	
TCMBank Lv et al. (2023) (https://TCMBank.cn/)	32529 diseases	TCM Database@Taiwan	Disease type, related target, external links: DisGeNET, MeSH, DO, HPO, UMLS	Relationship map (herb-component-target-disease)
CPMCP Sun et al. (2022) (http://cpmcp.top)	14434 diseases	MeSH, SIDER, UMLS, ChP-TCM, research on the standardization of TCM terminology, and pathology terminology standardization	Related component, related herb, related formula, related target, related disease/MM symptom/TCM symptom	Click on the uncomfortable body part to filter for symptoms that may match
	1148 MM symptoms			
	2285 TCM symptoms			

MM, modern medicine; DO, disease-ontology; UMLS, unified medical language system; ChP-TCM, pharmacopoeia of the people’s republic of China; TCM, traditional Chinese medicine.

The relationship between a target and the corresponding phenotype serves as a crucial bridge connecting a biological mechanism to the pathological manifestation in the human body. This connection was first established through the discovery of mutation-phenotype relationships. OMIM is a comprehensive repository that focuses on genetic and phenotypic data and interrelationships between the two. This database plays a pivotal role in naming and categorizing genetic phenotypes, thereby exerting a significant influence on the field of genetics (Funk et al., 2022). With the advent of the post-genomic era, the goal of deciphering the biological functions of target has evolved into the larger goal of delineating the intricate relationships between multiple genes and phenotypes. HPO and DisGeNET are comprehensive databases for analyzing and interpreting human gene-disease networks (Piñero et al., 2017; Köhler et al., 2021, 1). CPMCP and SymMap also include both TCM symptoms and modern medicine (MM) symptoms in an attempt to bridge TCM and modern medicine-based research through symptom associations (Wu et al., 2019; Sun et al., 2022).

## 7 ZHENG database

TCM involves a unique, intuitive understanding of physiological states. *ZHENG* differentiation and treatment (辩证论治) is the fundamental approach guiding clinical practice in TCM. Diagnoses and treatments are made by taking into account the individual differences between patients (Zhou et al., 2014; Wang and Zhang, 2017). *ZHENG* is a summary of the pathological and physiological discrepancies at each stage of a disease. It is also determined by factors such as the disease site and the nature of the disease (Wang et al., 2022a). TCM practitioners prescribe different formulas based on the *ZHENG* to achieve therapeutic efficacy. Explorations of *ZHENG*-formula and *ZHENG*-phenotype relationships represent a challenging area of research in both clinical practice and foundational TCM studies. A *ZHENG* database, SoFDA, has been constructed to record and collect *ZHENG* data (Zhang et al., 2022b). It includes both macroscopic data, such as ZHENG, phenotypes, and TCM formulas, and microscopic data (molecular mechanisms). Such databases promote a deeper understanding of ancient systematic medicine, TCM, and modern medicine. SoFDA implements two common association measures (Jaccard and Cosine similarity) to quantify relationships between clinical entities (e.g., *ZHENG*, phenotypes, and formulas). This allows users to compute the degree of indirect associations between the three entities in terms of six shared features: symptoms, genes, enriched gene ontology (GO) terms, enriched pathways, network modules, and network density. However, databases specifically focused on *ZHENG* are currently limited in number, and there are few comprehensive phenotypes analyses related to *ZHENG*.

## 8 Discussion

### 8.1 The emergence of network analysis triggered a surge in data generation and database construction

Database evolution is closely linked with current research trends and challenges over time (Sorokina and Steinbeck, 2020). Beyond serving as robust repositories for vast amounts of data, databases related to TCM systems pharmacology also represent pivotal milestones in summarizing the alternations of states in the TCM research. In this review, we retrospectively trace databases pertinent to computational analyses in TCM. Our primary focus is the detailed exploration and comparison of data structures within databases containing formula, herb, component, target, phenotype, and *ZHENG* data (Figure 1). Additionally, we delve into the intricate relationships between these entities within relevant databases. Systems biology is the cornerstone in the establishment of databases related to TCM systems pharmacology. It was until the emergence of component-target databases such as BindingDB, which summarize a large number of component-target relationships based on experimental data, allow mathematical simulation of component-target relationships, effectively addressing the challenge of identifying targets for numerous components (Gilson et al., 2016; Mendez et al., 2019). The emergence of component-target relationships as an area of study has bridged the gap between TCM and biological systems. In 2007, Yildirim et al. applied the principles of network biology by integrating and analyzing drug-gene and drug-protein interaction data. Their work revealed that the majority of drugs exert their effects through indirect modulation rather than direct targeting of disease-associated proteins (Csermely et al., 2005). Building upon this foundation, Hopkins proposed the research methodology of network analysis in pharmacology. He posited that drugs act on multiple targets and demonstrated enhanced efficacy and reduced toxicity through interactions among these multiple targets (Hopkins, 2008). The field of network analysis, which answers research questions from an inherently integrated standpoint, coincides remarkably well with the fundamental principles of TCM (Li and Zhang, 2013; Wang et al., 2021b). Over the course of Chinese history, thousands of herbs and over 300,000 formulas have been applied as medicines (Li et al., 2008). Often, the certain single herb appears in multiple formulas, each of which yields a distinct effect (Wang et al., 2021d). Compared to commercially available synthetic drugs, herbs exhibit a larger quantity of components with higher complexity. Consequently, there is a greater need to collect and organize information to uncover the patterns associated with herbal combinations and their therapeutic effects. Possibly driven by this rationale, the TCMID database was launched in 2012, including relationships between and among formulas, herbs, components, targets, and phenotypes. The inception of this database marked the emergence of the core chain of pharmacological research using herb (Xue et al., 2012; Huang et al., 2018). Subsequent databases related to TCM systems pharmacology have largely promoted establishment of relationships between and among the same entities. However, these newer databases have also offered enhanced capabilities for computational analyses.

**FIGURE 2:**
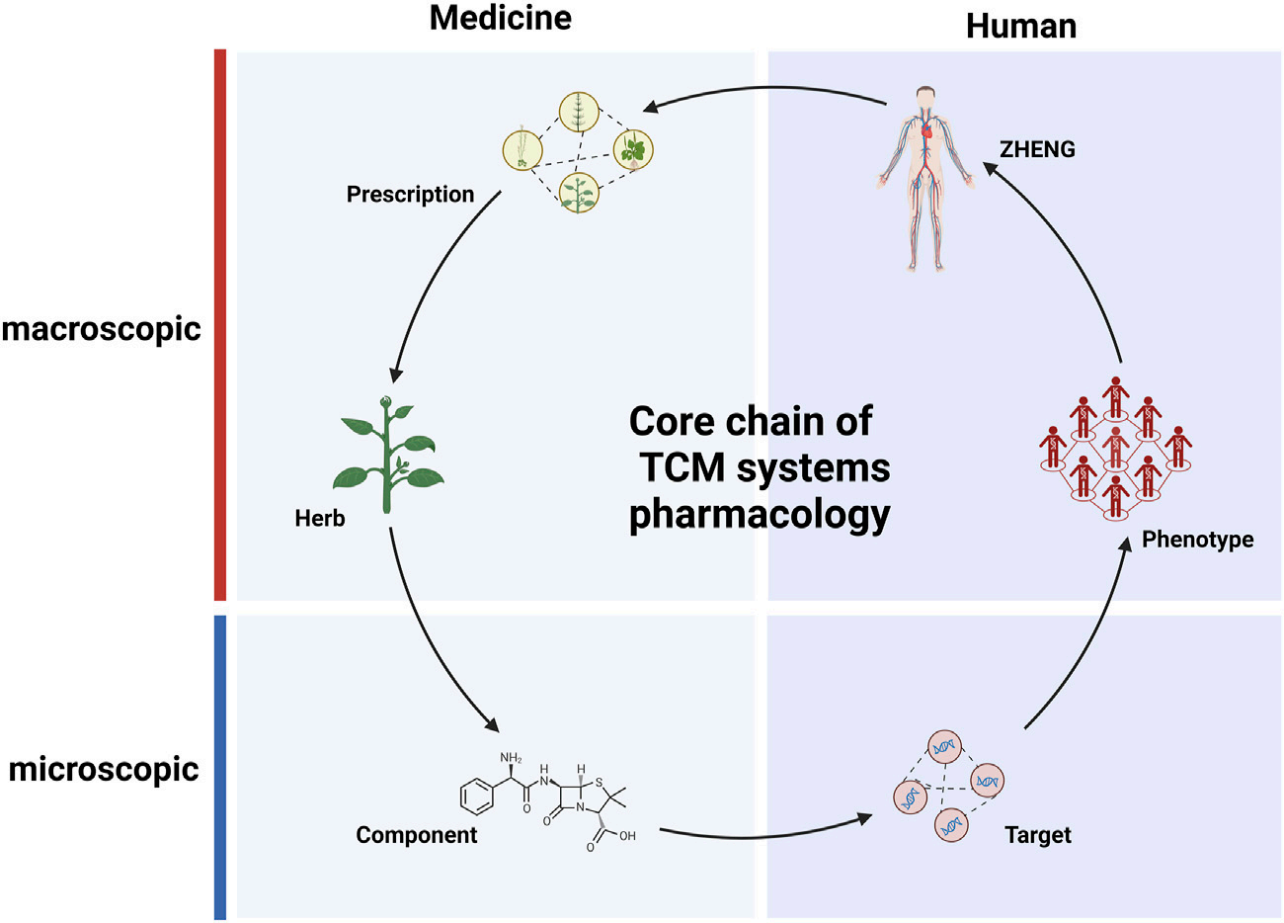
The central chain of pharmacological research in traditional Chinese medicine (TCM). Solid arrows represent primary relationships between entities within various hierarchical databases related to TCM.

In TCM, the stable coexistence of various clinical manifestations is defined as *ZHENG*, which is also the integrative description on the current status of complex biological system (Tang et al., 2008). Empirical explorations in TCM focus on establishing direct relationships between formulas and *ZHENG*. For patients sharing common pathological characteristics, TCM practice calls for the use of similar but not entirely identical formulas (Wang et al., 202a). Diagnosing and treating patients based on *ZHENG* differentiation can enhance the clinical effectiveness of the treatments. The integration of *ZHENG* and modern personalized medicine approaches could serve as a breakthrough for addressing current challenges in medical practice (He et al., 2008; Su et al., 2012; Chen et al., 2013). A significant amount of omics research is employed to uncover the physiological mechanisms of patients with different *ZHENG* (Wu et al., 2021; Akhoundova and Rubin, 2022). Experimental studies have revealed that patients with different *ZHENG*, but the same disease exhibit distinct biomarkers (Shang et al., 2022). The accumulation of this data can provide more accurate features for computational analysis of *ZHENG*. The rapid development of AI has enabled the training with and analysis of large datasets and led to advancements in personalized medicine. AI has been utilized to learn from tongue images and clinical diagnostic information, aiding in clinical diagnosis (Kanawong et al., 2012; Tang et al., 2021; Chen and He, 2022). Several computational studies have described the use of information about relationships between targets, phenotypes, and symptoms to recommend appropriate clinical formulas (Li et al., 2007; Kanawong et al., 2012; Zhou et al., 2014). It has become possible to reveal the essence of *ZHENG* based on a wealth of information, including phenotypes, and AI model. It thus appears that the process through which herbs exert their therapeutic effects follows the core formula-herb-component-target-phenotype-*ZHENG* chain (Figure 2). In fact, this core relationship chain built with extensive data may aid us in exploring from one entity to another, e.g., starting from a drug entity to explore its clinical applications, it offers an approach to uncover new clinical uses of existing drugs, thereby expanding our understanding and utilization of pharmaceutical resources. Similarly, application in an opposite direction is recommending personalized medications based on clinical phenotype entity. This interconnectivity, grounded in large-scale data, provides a robust framework for enhanced drug discovery and personalized medicine. It enables the identification of tailored therapeutic solutions catering to individual patient’s unique clinical presentations and needs (Zhang et al., 2019b). Indeed, there have been studies that utilize such relationship chains for recommendation of personalized medication. Researchers have created gene expression profiles for 189 diseases, then analyzed the perturbation characteristics of herbs based on the herb-component-target relationships within the database. Finally, they predicted the optimal combinations of herbs for treating diseases based on the mapping relationship between herbs and diseases (Chen et al., 2018). However, these studies are still in their early stages of research, and a substantial number of experiments are needed before they can be applied in clinical research.

Pharmacological research is concerned with the process of regulating biological systems through medications; TCM employs complex chemical systems to regulate biological systems (Liang et al., 2012). TCM research is based on the accumulation of extensive clinical experience, through which numerous associations between specific *ZHENG* and corresponding formula are established (Yang et al., 2020). Patients are primarily differentiated into subtypes to enable selection of suitable formulas (Wang et al., 2021c). Another aspect of TCM research involves discovering the efficacy of specific herbs for particular phenotypes. This allows for the incorporation of “specific herbs” into formulas, which are modified accordingly to address a specific patient’s disease state beyond the corresponding *ZHENG*. However, there is a lack of necessary research into the material bases and action mechanisms of formulas and herbs metioned above (Xu et al., 2019). This limitation has confined the development of new medical knowledge to the accumulation and extension of clinical experience. In contrast, modern medicine, which is based on chemistry and molecular biology, has be used to elucidate chemicalstructures, functions, and targets, providing modern pharmacology with an extremely precise perspective at the micro scale (Penrod et al., 2011; Zeeshan et al., 2020). However, the rate of new chemical drug production is slowing (Sadybekov and Katritch, 2023). During the development of modern pharmacology, a plethora of component-target relationships have been established (Santos et al., 2017). The establishment of these relationships has provided TCM research with numerous paradigms and methods. This, in turn, has endowed TCM pharmacology with the ability to unveil the formula systems regulating human biological systems, opening the “black box”. The core chain (formula-herb-component-target-phenotype-*ZHENG*) bridges the gap between macroscopic and microscopic levels; to some extent, it also explains the interactions between formula systems and the biological system at the molecular level (Figure 2). This framework has made it possible to conduct TCM systems pharmacology research.

### 8.2 Data accumulation encounters a research bottleneck in TCM computational pharmacology

To date, there has been a significant accumulation of data at various levels within the formula-herb-component-target-phenotype-*ZHENG* chain. The relationships between members of each level have been effectively organized and summarized in various databases. The research in TCM has leveraged the concept of networks, thereby advancing towards the approach with more characteristic’s systems science (Huang et al., 2017). However, in complex system, both formulas and modified herbal prescriptions are administered at specific quantities in practical applications, meaning that the herbal composition of formulas is quantitative, and the components within the herbs are quantitative (Luan et al., 2020). In the process of pharmacological research, the effective dosage of a drug is crucial and therefore carefully examined (Spencer and Jarvis, 1999). However, in the context of TCM databases and computational studies of TCM formulas, there are few quantitative calculations and little dosage information. Indeed, not only in TCM but also for active components in general, there have been few studies that provide absolute quantitative or relative quantitative (i.e., proportional) information. This approach raises doubts about the accuracy of computational predictions of the composition, efficacy, and mechanism of action associated with formulas. For instance, polyphenols could interact with multiple targets due to their unique nature of multiple hydrogen donor if not considering the effective concentration (Luca et al., 2020), but not all drug-target relationships identified through these methods necessarily translate into therapeutic effect, which presents one of the major limitations in *in silico* research. However, predictive research should apply “quantitative algorithm” to calculate the inhibition rate to the very target but not component-target relationship only; second, components must accumulate to a sufficient concentration around the target in cellular and animal experiments after passing through the cell membrane or even gastrointestinal tract and liver (Manukyan et al., 2019; Luca et al., 2020; Khojah et al., 2021).

Pharmacological research in TCM necessitates both qualitative and quantitative investigations of relationships between parameters in the “formula-herb-component-target-phenotype-*ZHENG*” chain. The relationships among these entities are highly intricate, constituting not one-to-one but rather many-to-many relationships. This complexity is reminiscent of neural networks, which are characterized by extensive intricate connections (Ma et al., 2014). Quantitative studies can be likened to parameters such as weights and biases in a neural network (Lu et al., 2022). In a previous study, the introduction of a novel coefficient aimed to replicate the proportional quantities of components relative to the weight of an herb of interest within a specific formula (Chu et al., 2020). This coefficient also serves to evaluate the pharmacological impact of antiarrhythmic herbal medicine Xin Su Ning capsule across various pertinent biological pathways (Wang et al., 2019b). However, the complex network of quantitative information requires systematic collection in relevant databases to facilitate systems pharmacology research of herbs.

### 8.3 Upcoming paradigm shifts in TCM pharmacological research

TCM databases offer a wealth of foundational data for pharmacological analyses of complex systems (e.g., formulas). They play pivotal roles in accelerating TCM-based computational science and pharmaceutical research. Moreover, these databases are essential for deciphering the intricate relationships among entities in the formula-herb-component-target-phenotype-*ZHENG* core chain. At present, such databases are primarily used for data retrieval rather than aiding in the discovery of new drugs/formulas or novel pharmacological mechanisms. However, many researchers have begun harnessing the extensive relationships described in databases such as those discussed here to simulate complex formulas. This approach aids in exploration of herb combination patterns (Niu et al., 2023), development of innovative drugs (Li et al., 2010), identification of mechanisms of disease intervention through herb (Gan et al., 2023), and enhancement of clinical research (Zhao et al., 2015; Wang et al., 2021c).

The essence of formulas and medicinal plants is a mixture of compounds. Referring to a single compound, the functionalities of chemical components are determined by their structures (Xiong et al., 2022). Chemical drugs exhibit limited structural diversity and target just over 700 different proteins. The constrained coverage of this chemical space is insufficient to address all modulable or pathological physiological mechanisms that occur in human disease states (Lipinski and Hopkins, 2004; Reymond, 2015; Stocker et al., 2020). Natural products, which are often referred to as single components in TCM, are numerous and display a wide range of chemical structures (Lachance et al., 2012). This diversity enables them to target a broader spectrum of receptors (Lipinski, 2016). These component therefore represent a valuable repository of potential therapeutic agents (Li et al., 2008). To date, a substantial body of research on TCM formulas has identified the key active components and core mechanisms of action (Zhang et al., 2017a; Wang et al., 2019a; Xu et al., 2020). This information continues to be instrumental in aiding the development of combination drugs composed of multiple components. Once the relationships between a significant number of components combinations and their therapeutic effects are understood, it becomes possible to create new formulas consist of those components based on specific requirements (Keith et al., 2005). This approach can minimize issues related to drug quality control and reduce the costs associated with drug development.

Advances in deep learning and foundation model (Hamet and Tremblay, 2017; Du et al., 2021) indicate that it is increasingly feasible to simulate the complex network encompassing the core chain. The emergence of foundation models is expected to provide tools with precise computational capabilities and entirely new perspectives on pharmacological calculations (Du et al., 2021; Zeng et al., 2022a). Additionally, foundation model-based generative AI has shown immense disruptive potential across various industries, including healthcare and medicine (Singhal et al., 2023b; Xiong et al., 2023). Currently, generative foundation models and medical models fine-tuned based on them have demonstrated strong general capabilities in many medical tasks (Singhal et al., 2023a). They have shown preliminary potential to simulate the corresponding relationships between the entities within the core chain. In the medical field, foundation models can be leveraged to perform various types of tasks, such as extracting key information from electronic health records and analyzing patient symptoms to make disease diagnoses (Xiong et al., 2023). These models can assist in automating data extraction and standardization procedures, leading to a substantial reduction in the time required to establish comprehensive medical databases (Singhal et al., 2023a). The integration of databases related to TCM with foundation models hold significant potential for establishing a knowledge graph in the field of TCM system pharmacology. This integration can enable the generation of knowledge graphs that encompass the relationships between various entities of TCM, formulas, herbs, *ZHENG*, and their pharmacological effects. It can facilitate the development of a question-and-answer system that provides relevant analytical solutions. Furthermore, the expansion of relevant data relationship dimensions in the systems pharmacology database may enhance the depth of computation in foundation models and improve the accuracy of computation. Using foundation model, a dataset comprising a substantial number of effective formulas and the corresponding phenotypes could be used to elucidate the intricate relationships among the entities of the core chain.

In summary, research on databases has made significant and substantial progress in recent times. A vast amount of data related to formula, herb, *ZHENG*, and diseases has been accumulated. A core chain of interrelated relationships has been established, linking the research entities. Furthermore, computational methods are now being employed to simulate and analyze the relationships between entities within this core chain. Currently, while there isn't a single database that can provide computational services to model the complex relationships among all entities in the core chain mentioned above, it's anticipated that with the advancement of technology, this stage is not too far off in the future. However, regarding the existing entities and relationships within the core chain, there are still numerous significant issues that cannot be overlooked. The presence of these issues poses a potential risk of failure in future computational pharmacological research (Sadybekov and Katritch, 2023). Most databases are interconnected resources, and even new databases are often updates or extensions of existing ones, with limited substantive changes to older data. An illustrative example is that some of these databases operate under the principle that if a specific chemical structure demonstrates activity, it's likely to have a similar effect on structurally similar sites. Therefore, we need more and better experiments for evaluation, and literature studies also contain many false positives/false negatives, so it's crucial to maintain clear data sources when incorporating them into the database. For the entire core chain, there is indeed the potential for quantitative calculations, which could enhance the rigor and accuracy of computational research in TCM. It's worth noting that there currently might not be corresponding databases or reports available to support this quantitative approach. It is hoped that in the future, databases will address this issue and foster greater collaboration between different domains, ultimately advancing the modernization and scientific exploration of TCM.
